# Phthisis and the Metropolitan Asylums Board

**Published:** 1905-04-29

**Authors:** 


### Phthisis and the Metropolitan Asylums Board.

The ever-present problem of what to do with the consumptive poor was again brought to the fore a few days ago, when the Metropolitan Asylums Board received a numerous and influential deputation, organised by the Metropolitan Branch of the Incorporated Society of Medical Officers of Health, urging the desirability of its undertaking the duty of providing sanatoria for the treatment of cases of consumption. The principal speakers on behalf of the deputation were Dr. Orme Dudfield, Medical Officer of Health for Kensington, Sir William Broadbent, M.D., and Sir Edmund Hay Currie, the Honorary Secretary of the Metropolitan Hospital Sunday Fund. The deputation desired to suggest that an Order of the Local Government Board should be obtained to make section 5 of the Metropolitan Poor Act, 1867, applicable to the case of "poor persons" suffering from pulmonary phthisis; and, if necessary, that an Act should be obtained to make the provisions of section 80 of the Public Health (London) Act, 1891, applicable to pulmonary phthisis as if such disease were therein mentioned as well as "fever, or small-pox, or diphtheria." Dr. Orme Dudfield, in supporting the memorial, did not dispute that the cost of carrying out its suggestions would be heavy, but strongly deprecated the erection of palaces, and urged the construction of the simplest possible suitable buildings, in which the expense of maintenance need not exceed 25s. per week per patient; so that, assuming 2,000 beds, which would enable the managers to deal with 4,000 cases a year, the total cost would be £130,000. If the Board would comply with the prayer of the memorial, he felt sanguine that the next forty years would see consumption far on the way to total extirpation. Sir William Broadbent, who followed, expressed his belief in the "probability that a large proportion of the sufferers would be restored permanently to health by sanatorium treatment." He added, however, that the persons admitted must be placed under definite restraint, for that it would not do "to receive patients and allow them to leave just when they liked, and thus, by returning prematurely to their homes and work, to waste the money which had been spent on them." In other words, it would be necessary to extend to a chronic disease like phthisis the restrictions upon personal liberty which can be enforced without difficulty during the comparatively short duration of the acute infections—such as small-pox or scarlet fever.

It will hardly be a matter for surprise that Mr. Scovell, the Chairman of the Metropolitan Asylums Board, adopted a tone of extreme caution in his reply. He put the whole matter in a nutshell by declaring it to be a question "whether the public and ratepayers of London were sufficiently educated and acquainted with the great wants of the tuberculous portion of the population to induce them to give a ready assent to the payment of increased rates for the purpose of providing sanatoria." He was quite sympathetic, but pointed out that the managers had no mandate to spend or to suggest expenditure in new directions, and said that they desired to throw the onus of inquiry upon the Local Government Board, and to receive from that source some guidance upon the subject. In these circumstances it is manifest that no action of the kind desired by the deputation can be taken without considerable delay, and, in the meanwhile, the question at issue is one which must be regarded from several points of view. It must not be forgotten that the mortality from phthisis, serious as it undoubtedly is, has for some years been steadily diminishing, and that, if the progress in this direction during the next forty years be anything like what it has been during the last, Dr. Dudfield's expectations will probably be fulfilled without the intervention which he seeks. It must also be remembered that the restrictions upon the liberty of the subject, which would be necessary in order to render sanatoria effective, would practically amount to sentences of prolonged imprisonment, upon medical authority, in the cases of persons who would ostensibly have little or nothing the matter with them, and who would frequently be rendered insubordinate by their desire to return to their normal surroundings. Their complaints would give rise, before long, to a flourishing new form of "anti-ism," to an "Anti-Imprisonment for Alleged Consumption Society," through which a great many unscrupulous persons would earn comfortable livings by denouncing the medical profession. Finally, with the greatest respect for Sir William Broadbent, we would venture to suggest that his expectation of permanent cures among the poor, as consequences of sanatorium treatment, might possibly not be fully realised in practice.

